# An *in vitro* ADME and *in vivo* Pharmacokinetic Study of Novel TB-Active Decoquinate Derivatives

**DOI:** 10.3389/fphar.2019.00120

**Published:** 2019-02-18

**Authors:** Lloyd Tanner, Richard K. Haynes, Lubbe Wiesner

**Affiliations:** ^1^Division of Clinical Pharmacology, Department of Medicine, University of Cape Town, Cape Town, South Africa; ^2^Centre of Excellence for Pharmaceutical Sciences, Faculty of Health Sciences, North-West University, Potchefstroom, South Africa

**Keywords:** pharmacokinetics, ADME, tuberculosis, anti-TB chemotherapy, decoquinate, DMPK, LC-MS/MS

## Abstract

Tuberculosis (TB) is currently the leading cause of mortality due to an infectious disease, despite the existence of multiple effective first-line and second-line drugs. The current anti-TB regimen requires a prolonged treatment period of around 6 months and is only efficacious against drug-sensitive strains of *Mycobacterium tuberculosis* (*Mtb*). With a rise in cases of multi-drug resistant and extensively drug resistant strains of *Mtb*, newer treatments comprising compounds with novel mechanisms of action are required. Although decoquinate (DQ) is inactive against *Mtb*, its derivatives are of interest to anti-TB drug discovery because of their potential to permeate the mycobacterial cell wall, *Mtb*-infected macrophages, and granulomatous lesions by passive diffusion. The compounds also display mechanisms of action which are unlike those of currently used quinolones, potentially displaying activity against new targets. Three such derivatives bearing an alkyl group at N-1 and an amide group at C-3 (RMB 041, -043, and -073) displayed potent *in vitro* activities against *Mtb* H37Rv (90% minimum inhibitory concentrations, MIC90 = 1.61, 4.18, and 1.88 μM, respectively) and high selectivity indices (10–25). In this study, we evaluated the drug-like properties (*in vitro* microsomal stability, microsomal/plasma protein binding, kinetic solubility, lipophilicity, and passive permeability) and pharmacokinetic (PK) parameters of these compounds after intravenous and oral administration to male C57BL/6 mice. The compounds showed markedly improved kinetic solubilities compared to that of the parental DQ and were metabolically stable *in vitro*. The maximum concentrations reached after oral administration were 5.4 ± 0.40, 5.6 ± 1.40, and 2.0 ± 0.03 μM; elimination half-lives were 23.4 ± 2.50, 6.2 ± 0.80, and 11.6 ± 1.30 h; and bioavailabilities were 21.4 ± 1.0, 22.1 ± 2.2, and 5.9 ± 1.3 for RMB041, -043, and -073, respectively. These compounds therefore display promising drug-like properties, and their PK/toxicity profiles (including long half-lives both *in vitro* and *in vivo*) support their potential as candidates for further investigation in animal models of *Mtb* infection.

## Introduction

The resurgence of tuberculosis (TB), an infectious disease caused by *Mycobacterium tuberculosis* (*Mtb*), is partly due to the rise of the human immunodeficiency virus/acquired immune deficiency syndrome (HIV/AIDS). Although its incidence is slowly decreasing in response to the implementation of multi-drug treatment regimens, TB continues to progress in numerous populations, with an estimated 10 million new infections and 1.3 million deaths in 2017 [World Health Organization (WHO), 2018]. The World Health Organization (WHO) has recommended four first-line drugs [isoniazid (INH), rifampicin (RIF), ethambutol (EMB), and pyrazinamide (PZA)] and various second-line drugs for the treatment of TB. *Mtb* resistance to first-line medications is increasing, and novel therapeutic agents and drug combinations are thus urgently required (Dheda et al., 2017).

To this end, we have assessed new combinations of drugs with both oxidant and redox properties coupled with a third partner drug, with the initial focus on quinolone derivatives (Beteck et al., 2018; Morake et al., 2018; Haynes^1^). Since the discovery of the antibiotic activity of the first quinolone nalidixic acid in 1962, quinolones have been used to treat a variety of pulmonary infectious diseases (Andriole, 2005). Newer quinolones with substituents at the C-6, -7, and -8 positions were developed to act on mycobacterial DNA gyrase and topoisomerase IV (Onodera et al., 2001; Aubry et al., 2004). Fluoroquinolones bearing fluorine at C-6 are active against *Mtb* and have been included in anti-TB treatment regimens since 1984 (Gay et al., 1984). However, the recent emergence of fluoroquinolone resistance has highlighted the need for novel quinolone structures [Gillespie et al., 2014; World Health Organization (WHO), 2016].

**FIGURE 1:**
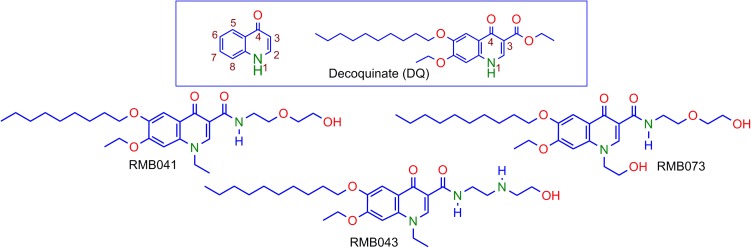
Numbering of the quinolone scaffold and structure of decoquinate (DQ) (box), and the DQ derivatives RMB041, RMB043, and RMB073.

Decoquinate (DQ) (6-decoxy-7-ethoxy-4-oxo-1*H*-quinoline-3-carboxylic acid ethyl ester, DQ; Figure 1), an anticoccidial quinolone, has been used in poultry feed for over 50 years [European Food Safety Authority (EFSA), 2003; Taylor and Bartram, 2012]. Although DQ is inactive against *Mtb*, the lipophilic decyl side chain of the derivatives should allow permeation through the mycolic acid cell wall of *Mtb*, infected macrophages, and granulomatous lesions containing *Mtb* by passive diffusion (Hurdle et al., 2011; Chen et al., 2018). However, DQ shows poor drug-like properties, including low solubility in water (0.06 mg L^-1^) and in aqueous buffer at pH 4.9 (<0.01 mg L^-1^) [European Food Safety Authority (EFSA), 2003]. Thus, DQ was converted into derivatives in which the ethyl ester group was replaced by the more polar, less readily metabolized amide, and alkyl groups replaced the quinolone H atom at N-1 (Beteck et al., 2016). The relatively soluble DQ amide derivatives RMB041, RMB043, and RMB073 incorporating ethanolamino- or ethylenediamine-linked amide side chains and an ethyl or 2’-hydroxyethyl group at N-1 were identified as hit compounds with *in vitro* activities against *Mtb* H37Rv (90% minimum inhibitory concentrations (MIC_90_) = 1.61, 4.18, and 1.88 μM for RMB041, -043, and -073, respectively), whereas other simple amides derived from DQ were inactive against *Mtb* (Beteck et al., 2018). All compounds displayed high selectivity for *Mtb* [selectivity indices (SI): 10–25] as revealed by *in vitro* cytotoxicity studies against Chinese hamster ovarian (CHO) cell-lines (IC_50_ = 20.0, 80.0, and 33.9 μM for RMB041, -043, and -073, respectively). Preliminary mechanism of action studies exclude mycobacterial respiration, but indicate cell wall homeostasis as a likely target, as well as late-stage effects on DNA metabolism, (Beteck et al., 2018) which are targets different to those of currently used fluoroquinolones. RMB041 shows similar *in vitro* potency to that of ciprofloxacin (MIC_90_ 1.5–12 μM), gatifloxacin (0.66–1.3 μM), and moxifloxacin (0.62–1.3 μM) (Ginsburg et al., 2003).

Before performing costly and challenging *in vivo* efficacy studies required to develop new TB drugs, (Franzblau et al., 2012; Dartois and Barry, 2013; Nuermberger et al., 2016) the compounds’ drug-like properties must be determined. Therefore, the *in vitro* absorption, distribution, metabolism, and excretion (ADME) were assessed, including kinetic solubility, microsomal stability, passive membrane permeability, lipophilicity, and plasma/microsomal protein binding. The *in vivo* pharmacokinetic (PK) properties of the compounds were also assessed in a mouse model. To check the consistency of the methods used, compound behavior in mouse whole-blood and compound stability under the various assay conditions were also investigated.

## Materials and Methods

### Ethics Statement

All animal studies were conducted with approval from the Animal Ethics Committee of the University of Cape Town (013/032). The experiments were conducted in accordance with the National Code for Animal Use in South Africa (National Research Council, 2011). The use of human plasma was approved by the Human Research Ethics Committee of the University of Cape Town (HREC 783/2016).

### Materials

Compounds RMB041, -043, and -073 were prepared and purified [purity ≥ 96%, determined via high-performance liquid chromatography (HPLC)] (Beteck et al., 2018). Human plasma was obtained from the Western Province blood transfusion services (Cape Town, South Africa). Potassium dihydrogen phosphate and dipotassium hydrogen phosphate were purchased from Merck (Darmstadt, Germany). Analytical-grade acetonitrile (ACN) was purchased from Anatech (Johannesburg, South Africa). Analytical-grade dimethyl sulfoxide (DMSO), formic acid (FA), carbamazepine, propranolol hydrochloride, warfarin, procaine hydrochloride, and vinpocetine were obtained from Sigma-Aldrich (St. Louis, MO, United States). Water was purified via a Milli-Q purification system (Millipore, Bedford, MA, United States). Liver microsomes were obtained from Xenotech (Kansas City, KS, United States).

### ADME Assays

All the *in vitro* ADME assays presented below were completed with the inclusion of control compounds (data not shown) to ensure that each assay performed optimally during this study.

#### Kinetic Solubility

Stock solutions of each compound prepared in DMSO (10 mM) were spiked in duplicate into phosphate buffer at pH 7.4 (final concentration = 200 μM). Calibration samples were prepared by spiking individual compounds in DMSO at 11, 100, and 220 μM. These were used to generate a calibration curve to determine compound concentrations in the test samples. The samples were agitated for 2 h using a plate shaker (500 rpm, 22°C). Approximately 150 μL of the resulting supernatant was transferred to a 96-well analysis plate and the calibration and test samples were analyzed via HPLC with a diode array detector (Agilent 1200 Rapid Resolution HPLC, Agilent Technologies, Santa Clara, CA, United States) comprising a reverse-phase Gemini-C18 analytical column (5 μm, 50 mm × 2 mm; Phenomenex, Torrance, CA, United States). Mobile phases comprised 0.1% FA in water (A) and 0.1% FA in ACN (B). The run lasted 3 min and a gradient elution method was used (Table 1).

**Table 1:** Gradient elution conditions for the kinetic solubility and lipophilicity assays.

Time (min)	% A (0.1% FA in water)	% B (0.1% FA in ACN)
0.00	100	0
0.20	100	0
1.40	0	100
1.58	0	100
1.60	100	0
3.00	100	0


#### Lipophilicity

Compound solutions (10 μL, 1 μg/mL) were added to 1-octanol and phosphate buffer (pH 7.4, 1:1 *v*/*v*) in a 96-well plate. The plate was agitated for 2 h at 22°C and 500 rpm. The buffer and 1-octanol layers were removed separately and transferred into 96-well plates for HPLC analysis, using the conditions and instrumentation described above for kinetic solubility. The different layers were analyzed to obtain a logD value.

#### Plasma and Microsomal Protein Binding

Compound solutions prepared in DMSO were diluted in phosphate buffer (final concentration = 1 μg/mL), and were spiked into human plasma and microsomal fractions (total volume = 1 mL). Aliquots were then transferred in duplicate to: (i) a final concentration plate that was immediately quenched with 23.6 ng/mL internal standard (IS, carbamazepine) in ACN, (ii) a degradation control, which was placed in a water bath at 37°C for 4 h, and (iii) ultracentrifuge tubes, which were centrifuged for 4 h at 37°C and 30 000 × *g*. All reactions were stopped by the addition of ACN containing 23.6 ng/mL carbamazepine. The samples were subjected to liquid chromatography-tandem mass spectrometry (LC-MS/MS) analysis on an AB Sciex 4000 Q Trap hybrid triple quadrupole linear ion-trap MS (AB Sciex, Framingham, MA, United States) coupled to an Agilent 1200 HPLC (Agilent) with a reverse-phase Gemini-C18 analytical column (5 μm, 50 mm × 2 mm; Phenomenex) at 35°C. Mobile phases comprised 0.1% FA in water, and 0.1% FA in ACN. The flow rate was 600 μL/min with a run time of 6 min. A gradient elution was employed (Table 2).

**Table 2:** Gradient elution conditions for the microsomal and protein binding, parallel artificial membrane permeability assay (PAMPA), and microsomal stability assays.

Time (min)	% A	% B
0.00	95	5
0.5	95	5
2.0	5	95
3.6	5	95
3.7	95	5
6.0	95	5


#### Parallel Artificial Membrane Permeability Assay (PAMPA)

The permeability of the compounds was assessed via a PAMPA using a 96-well multiscreen filter plate (0.4-μM pore size). The filter plate was coated with 5% hexadecane in hexane and was allowed to dry before starting the assay. Lucifer yellow was added to the apical side of the filter plate in each well containing test compound solutions prepared in DMSO. A solution of each compound was diluted into phosphate buffer at pH 7.4 (final concentration = 1 μg/mL) and was added to the apical portion of the donor plate. The acceptor plate was filled with blank buffer (pH 7.4). The plates were slotted into each other and agitated at 80 rpm for 4 h at 22°C. Samples from donor and theoretical equilibrium wells were matrix-matched with blank phosphate buffer at pH 7.4. Samples were treated with ACN containing IS (carbamazepine, 23.6 ng/mL) and were submitted for LC-MS/MS analysis on an Agilent Rapid Resolution HPLC and AB Sciex 4500 MS. A portion of the sample containing Lucifer yellow was analyzed on a BioRad iMark^TM^ Microplate Absorbance Reader (BioRad, Hercules, CA, United States; excitation 490 nm, emission 510–570 nm) to determine *P*_app_ (acceptable values < 50 nm/s) using Eqs 1–3 (Palm et al., 1999; Wohnsland and Faller, 2001; Jung et al., 2006).

**Equation 1. Lucifer yellow permeability value (*P*_app_)**

$$P_{\mathrm{app}}=C\;\times \;-\ln\left(1-\frac{Acceptor\;well_{absorbance}}{Donor\;well_{absorbance}}\right)$$

where, *C* is calculated using Eq. 2.

**Equation 2. Permeability factor (***C***)**

$$C=\frac{VD\;\times \;VA}{(VD+VA)\;\times \;A\;\times \;t}$$

where, *VA* is the volume of the acceptor compartment (0.25 cm^3^), *VD* is the volume of the donor compartment (0.15 cm^3^), *A* is the accessible filter area (0.024 cm^2^), and *t* is the incubation time (s). The peak areas of the samples were used to determine the *P*_app_ of each compound using Eq. 3.

**Equation 3. Compound permeability (*P*_app_)**

$$P_{app}=C\;\times \;\;-\ln\left(1-\frac{Acceptor\;well_{peak\;area}}{Donor\;well_{peak\;area}}\right)$$

where, *C* is calculated using Eq. 2.

#### Metabolic Stability

RMB041, -043, and -073 (0.1 M) prepared in DMSO were incubated separately with 530 μL mouse and human microsomes (0.4 mg/mL) at 37°C with the cofactor NADPH (1 mM). Compounds were assessed at 0 and 60 min. The reaction was stopped by the addition of ice-cold ACN containing IS (carbamazepine, 23.6 ng/mL). The samples were analyzed via LC-MS/MS using the instrumentation and conditions described above.

The *in vitro* half-life, intrinsic clearance rate, and hepatic extraction ratio were predicted using results from this assay in the following equations (Obach, 1999).

**Equation 4. Predicted *t*_1/2_**

$$\frac{V_{m}\;\times \;\;t\frac{1}{2}}{K_{\mathrm{Mapp}}}\;=\;ln2+\frac{0.5{\left[S\right]}_{t=0}}{K_{\mathrm{Mapp}}}$$

where, ln 2 ≥ $\frac{0.5[S]}{K_{\mathrm{Mapp}}}$ assuming one *t*_1/2_ has passed, *V*_m_ is the rate of maximum metabolism, [*S*] is the concentration of the substrate, and *K*_Mapp_ is the apparent rate of metabolism.

**Equation 5. *CL*_int_**

$$CL_{int}=\;\left(\frac{0.693}{t\frac{1}{2}\;(\min)}\right)\;\times \;\left(\frac{Volume\;ofincubation\;(\mu L)}{microsomal\;protein\;(\mu g)}\right)$$

where, *t*_1/2_ is calculated in min.

**Equation 6. Hepatic extraction ratio**

$$E_{H}=\frac{Fu\;\times \;CL_{int}}{QH+Fu\;\times \;CL_{int}}$$

where, E_H_ is hepatic extraction ratio, *Fu* is fraction of unbound drug in the plasma, and *QH* is blood-flow to the liver.

### *In vivo* Pharmacokinetics

#### Animals

Healthy male C57BL/6 mice, 12 to 16 weeks old, weighing approximately 30 g, were maintained at the animal facility of the University of Cape Town. Mice were fed a standard laboratory diet and water *ad libitum*. Mice were housed in 27 × 21 × 18 cm cages, under controlled environmental conditions (26 ± 1°C with 12-h light/dark cycles). Mice were acclimatized to their experimental environment for 4 days before the experiment started.

#### Oral Drug Administration and PK Sample Collection

Clear suspensions of RMB041, -043, and -073 were prepared in 100% hydroxypropyl methylcellulose and administered via oral gavage (20 mg/kg, volume = 200 μL, *n* = 3). Blood samples (20 μL) were collected on ice in heparinized microvials via tail bleeding at 0, 0.5, 1, 3, 5, 8, 10, 24, and 48 h following drug administration and stored at -80°C.

#### IV Administration and PK Sample Collection

Solutions for IV injection (5 mg/kg) were prepared using a mixture of dimethyl acetamide, polyethylene glycol, and polypropylene glycol (1:3:6, *v/v*) and were injected into the penile vein (80 μL) following anesthesia of mice with intraperitoneal injection (IP) of ketamine/xylazine (75–100 mg/kg + 10 mg/kg; *n* = 3). Blood samples were collected on ice in heparinized microvials via tail bleeding at 0.083, 0.5, 1, 3, 5, 8, 10, 24, and 48 h following drug administration and stored at -80°C.

#### Sample Processing

Mouse whole-blood samples (20 μL) were each treated with 100 μL ACN containing IS (carbamazepine, 1 μg/mL) to precipitate blood proteins, and were subjected to vigorous vortexing for 1 min and centrifugation at 5590 × *g* for 5 min. The supernatant layer containing compound (50 μL) was removed from each sample and added to the analysis plate. Thereafter, samples were dried down under nitrogen. A solution of ACN and water (1:1, *v*:*v*, 100 μL) was added to each well, and samples were analyzed via LC-MS/MS.

To determine compound concentrations in mouse whole-blood samples, seven calibration samples (range 0.980 to 4000 ng/mL), as well as low-, medium-, and high-concentration quality control (QC) samples (3, 1600, and 3200 ng/mL, respectively) were prepared in mouse blood and subjected to ACN precipitation as described above, in triplicate. Calibration, QC, and test samples were analyzed via LC-MS/MS. Calibration samples were used to plot standard curves, from which test sample concentrations were calculated via quadratic regression (weighting factor = 1/*x*).

#### LC-MS/MS Analysis

A reverse-phase HPLC column (Gemini NX, C18, 2.6 μm, 50 × 2.1 mm, Phenomenex) was used to separate the compounds and IS (mobile phase B, 0.1% FA in ACN; mobile phase A, 0.1% FA in analytical-grade water). The gradient used is listed in Table 2 and MS/MS settings are listed in Table 3. Auto-sampling was performed using an Agilent 1200 series auto-sampler. Data acquisition and evaluation were performed using the Analyst 1.6.2 software (Applied Biosystems, Foster City, CA, United States).

**Table 3:** MS/MS settings used for analysis of carbamazepine, RMB041, -043, and -073 in mouse whole-blood samples.

Parameter	RMB041	RMB043	RMB073	Carbamazepine
Protonated precursor ion (*m*/*z*)	505.2	505.4	521.4	237.1
Product ion (*m*/*z*)	400.2	477.2	416.1	194.1
Ion spray voltage (V)	5500	5500	5500	5500
Nebulizer gas (AU)	30	50	30	25
Curtain gas (AU)	20	20	20	30
Auxiliary gas (AU)	45	65	45	40
Source temperature (°C)	400	400	400	400


*AU, arbitrary unit.*

#### Data Analysis

Plots of concentration against time were used to determine the maximal drug concentration (*C*_max_), time at which *C*_max_ is reached (*T*_max_), area-under-the-curve from time zero to infinity (AUC_0-∞_), and the elimination half-life (*t*_1/2_). Using these values, the clearance, volume of distribution, and oral bioavailability (BA) were calculated using the non-compartmental analysis (NCA) software PK Solutions version 2.0 (Summit Research Services, Montrose, CO, United States).

#### Partial Method Validation

The LC-MS/MS method used was partially validated by assessing recovery from mouse whole-blood, bench-top stability (3 h), auto-sampler stability (48 h), formulation stability (oral and IV, 2 h), freeze-thaw stability (3 cycles), and matrix effects (details in Supplementary Material).

## Results

### ADME Properties

The compounds’ solubility, *t*_1/2_, intrinsic clearance rates (*CL*_int_), and hepatic extraction ratios (E_H_) are listed in Table 4. Solubility was >150 μM (upper limit of the assay) for all compounds. LogD values, which provide an indication of lipophilicity in an octanol/water partitioning assay, were 0.48, 0.84, and 0.36 for RMB041, -043, and -073, respectively.

**Table 4:** Predicted solubilities, lipophilicities, and microsomal stabilities *in vitro.*

Compound	Predicted solubility (μM)	Lipophilicity (LogD)	*t*_1/2_ (min)		*CL*_int_ (μL/min/mg)		E_H_	
					
			Human	Mouse	Human	Mouse	Human	Mouse
RMB041	>150	0.48	>150	>150	16.00	26.80	<0.43	<0.3
RMB043	>150	0.84	85	60	83	109.3	0.4	0.51
RMB073	>150	0.36	>150	>150	7.78	14.96	<0.43	<0.3


*t_*1/2*_, half-life; *CL*_*int*_, intrinsic liver clearance rate; E_*H*_, hepatic extraction ratio.*

Metabolic stability was assessed as the compound’s depletion from a starting concentration of 0.1 M over 1 h in mouse and human microsomal fractions. The *in vitro* half-life can be predicted from this assay. The linear regression from a graph relating natural logarithmic percentage of the compound remaining versus the incubation time was used to calculate the half-life, *CL*_int_, and E_H_.

The percentage plasma protein binding of RMB041, -043, and -073 was 89.6, 77.9, and 84.4%, respectively (Table 5). Microsomal protein binding was 94.7, 94.7, and 88.6%, respectively, and the compounds’ permeability values were -4.8, -4.8, and -4.5, respectively.

**Table 5:** Predicted *in vitro* protein and microsomal protein binding (fraction unbound) and predicted *in vitro* permeability values (Log *P*_app_).

	Protein binding		PAMPA
		
Compound	Plasma protein (fraction_unbound_)	Microsomal protein (fraction_unbound_)	Log *P*_app_
RMB041	0.1	0.06	-4.8
RMB043	0.23	0.06	-4.8
RMB073	0.16	0.11	-4.5


*PAMPA, passive membrane permeability assay.*

### LC-MS/MS Assay Performance

The assay used to analyze mouse whole-blood samples achieved a percentage accuracy of 99.1 to 112.7% for the seven calibration standards, and 99.8 to 104.5% for the QCs. The calibration curves were best fitted with quadratic regression using a normalized peak area (compound/IS) against the x-axis (concentration in samples), with weighting factor 1/*x*. The lower limit of quantitation for calibration curve samples was 0.98 ng/mL for all three compounds, and the correlation coefficients for all curves were ≥0.99.

### *In vivo* Pharmacokinetics

**FIGURE 2:**
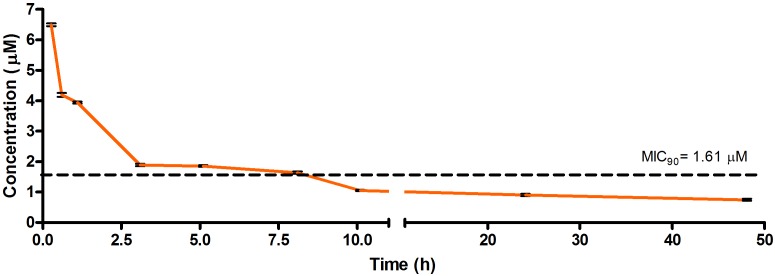
Total concentrations for RMB041 following intravenous administration in an uninfected murine model.

**FIGURE 3:**
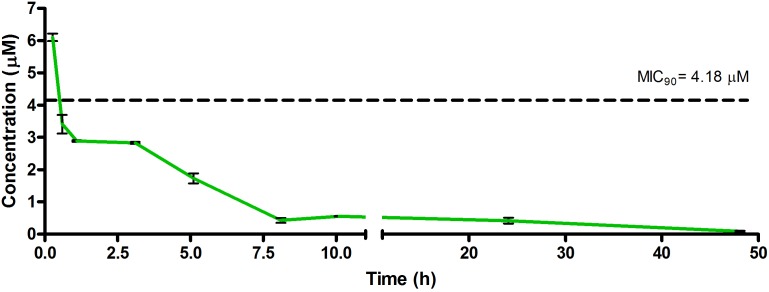
Total concentrations for RMB043 following intravenous administration in an uninfected murine model.

**FIGURE 4:**
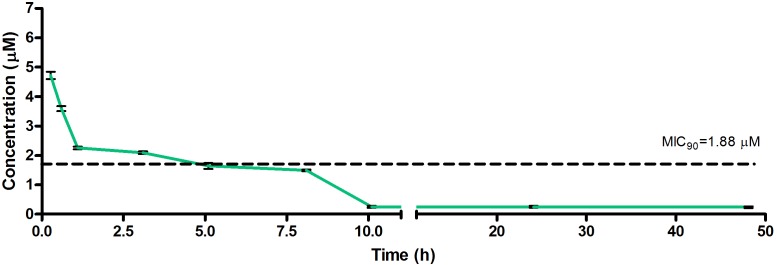
Total concentrations for RMB073 following intravenous administration in an uninfected murine model.

**FIGURE 5:**
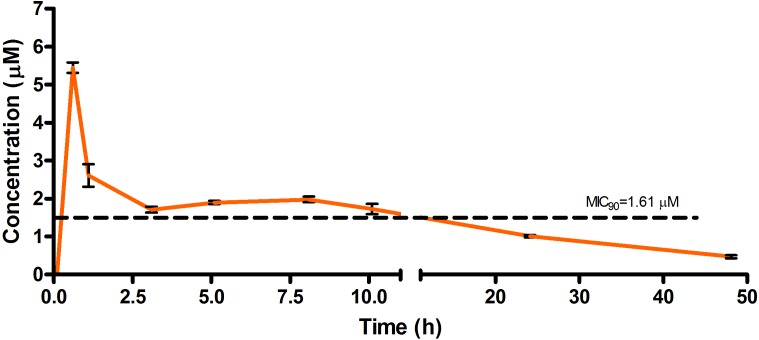
Total concentrations for RMB041 following oral administration in an uninfected murine model.

**FIGURE 6:**
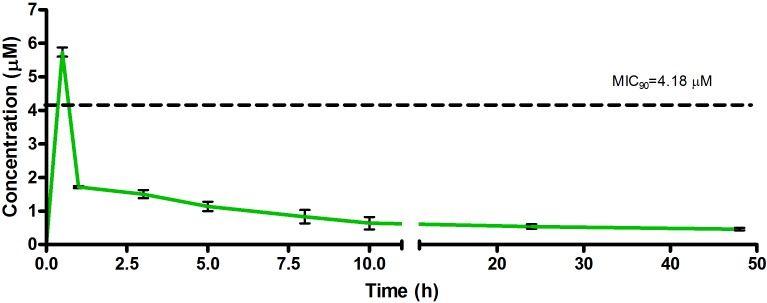
Total concentrations for RMB043 following oral administration in an uninfected murine model.

**FIGURE 7:**
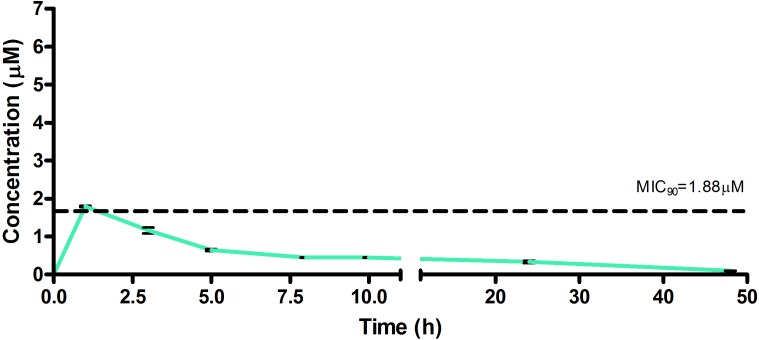
Total concentrations for RMB073 following oral administration in an uninfected murine model.

The drug concentrations indicated in Figure 2–7 were used to calculate the PK parameters in Table 6 via NCA. Here, the area under the murine whole-blood concentration-time graph is divided into sequential trapezoids that are summed to determine the AUC, which is used to determine *t*_1/2_, apparent volume of distribution (Vd), clearance, and percentage BA.

**Table 6:** Pharmacokinetic parameters from murine experiments with standard deviations shown for each value (*n* = 3).

Compound	*t*_1/2_ (h)	*T*_max_ (h)	*C*_max_ (μM)	Vd (L/kg)	Cl_tot_ (mL/h/kg)	AUC (min μmol/L)	Bio-availability (%)
IV (*n* = 3)							
RMB041	62.3 ± 6.7	-	-	1.2 ± 0.03	23.1 ± 0.3	29250.4 ± 309.0	-
RMB043	8.6 ± 0.4	-	-	4.6 ± 1.6	70.5 ± 4.2	10068.4 ± 127.8	-
RMB073	15.3 ± 3.2	-	-	3.9 ± 0.5	34.5 ± 1.3	15940.0 ± 400.0	-
Oral (*n* = 3)							
RMB041	23.4 ± 2.5	0.5	5.4 ± 0.4	-	-	25012.2 ± 1081.0	21.4 ± 1.0
RMB043	6.2 ± 0.8	0.5	5.6 ± 1.4	-	-	8915.7 ± 1017.0	22.1 ± 2.2
RMB073	11.6 ± 1.3	0.5	2.0 ± 0.03	-	-	3771.0 ± 298.0	5.9 ± 1.3


*t_*1/2*_, half-life; *T*_*max*_, time to maximum concentration; *C*_*max*_, maximum concentration; Vd, apparent volume of distribution; Cl_*tot*_, calculated total clearance; AUC, area under the curve.*

The total concentration versus time achieved with the IV formulations are displayed in Figure 2–4 with compound concentrations compared to their respective *in vitro* TB MIC_90_ concentrations. Standard deviations were used to generate error bars for each time point (*n* = 3).

The total concentration versus time achieved with the oral formulations are displayed in Figure 5–7 with compound concentrations compared to their respective *in vitro* TB MIC_90_ concentrations. Standard deviations were used to generate error bars for each time point (*n* = 3).

## Discussion

The DQ derivatives RMB041, -043, and -073 (Figure 1) showed high *in vitro* activities and selectivity for *Mtb* (SI: 10–25) (Beteck et al., 2018). These hit compounds were subjected to *in vitro* ADME assays, and displayed high solubility (>150 μM). DQ itself shows low aqueous solubility in water (<0.0001 μM) and consequently, low gastrointestinal absorption, which has limited its development as a therapeutic agent (Beteck et al., 2016). The derivatives here, show greater solubility potentially improving gastrointestinal absorption.

The *in vitro t*_1/2_ of RMB041 and -073 (>150 min), as determined by rates of microsomal degradation, were relatively long (Table 4), whilst that of RMB043 was lower (<100 min). The *CL*_int_ was low for RMB073 and -041 (<20 μL/min/mg), and was higher for RMB043 (<110 μL/min/mg). RMB 041 and -073 performed well compared to EMB (>75 μL/min/mg) and INH (<22 μL/min/mg) but less so in comparison to PA-824 and RIF (both <10 μL/min/mg) (Lakshminarayana et al., 2015). Determination of *in vitro CL*_int_ can help to identify whether the primary route of clearance is metabolism or whether the drug is eliminated unchanged. *CL*_int_ also allows compounds ranking for further *in vitro* assays and dosing calculations for clinical trials (Obach, 1999; Naritomi et al., 2001).

RMB041 and -043 demonstrated log *P*_app_ values of -4.8, while RMB073 showed higher permeability (-4.5). Compounds with log *P*_app_ values > -5 are considered highly permeable while those with values <-5 have low permeability (Wohnsland and Faller, 2001). The long, flexible alkyl chains may allow compounds to permeate cell membranes and mycolic cell wall of *Mtb*. The moderate to high plasma and microsomal protein binding of all RMB compounds indicate lower unbound drug fractions in the circulatory system.

Partial validation of the LC-MS/MS method involved assessment of the recovery, benchtop stability, freeze-thaw stability, autosampler stability, and formulation stability (full details in the Supplementary Information). The recovery of the compounds was >90% and the results are consistent, precise, and reproducible, as shown here (Supplementary Tables S1–S3) [Food and Drug Administration (FDA), 2018].

The calculated elimination *t*_1/2_ after IV administration of RMB041, -043, and -073 were 62.3 ± 6.73, 8.6 ± 0.40, and 15.3 ± 3.20 min, respectively (Table 6). These represent relatively long *t*_1/2_ when compared with those of other drugs such as RIF (7.19 h ± 0.42) (Ji et al., 1993; Lyons et al., 2013), INH (1.7 h ± 0.17) (Grosset and Ji, 1998; Jayaram et al., 2004), PZA (1.05 h ± 0.14) (Grosset and Ji, 1998), bedaquiline (53.00 h ± 6.00) (Andries et al., 2005; Rouan et al., 2012), clofazimine (87.46 h) (Swanson et al., 2015), and moxifloxacin (1.3 h) (Siefert et al., 1999).

PK data for known TB drugs are derived from *Mtb*-infected murine models, potentially negating a direct comparison between these results and ours. However, the uninfected murine model offers great value in translating *in vitro*-determined ADME properties into an *in vivo* model in a biosafety level (BSL) II environment. Early identification of compounds with high Vd or *t*_1/2_ values is essential to developing targeted drugs that are able to penetrate into the complex granuloma environment (Dartois and Barry, 2013; Dartois, 2014).

The *in vivo t*_1/2_, although considered medium to long, were shorter than predicted by the *in vitro* data (Obach, 1999). Drugs with *t*_1/2_ longer than those of current TB drugs may hold the key to shortening the duration of TB treatment (Warner and Mizrahi, 2014). This would reduce the cost of current therapies and improve treatment adherence in low-resource settings (Zager and McNerney, 2008; Dheda et al., 2017). These compounds showed low clearance rates *in vitro*. When considering the moderate to high solubility and high permeability, this indicates high exposures, *t*_1/2_, and BA for these compounds.

The oral BA in mice was >20% for RMB041 and -043, and >5% for RMB073, which is substantially longer than that of the parent molecule DQ (Beteck et al., 2016). Efforts have already been made by other groups to enhance the *in vivo* efficacy of DQ using nanoparticle formulation (Wang et al., 2013). However, our rationale was to synthesize stable and tractable DQ derivatives that can be developed further as potential anti-TB agents. Although all three compounds have comparably favorable *in vitro* ADME properties, the longer *in vivo* half-life and higher bioavailability of RMB041 compared to those of the other compounds make it the preferred compound to be taken forward for further studies. RMB043 and -073 provide excellent back up compounds should any cardiac toxicity or other factors affect the progression of RMB 041.

Future experiments to determine murine *in vivo* efficacy and whether compounds are able to penetrate into the organs in which *Mtb* resides (Takai et al., 2014) are required. To be considered as a suitable drug, the compound should distribute to the lungs, the site of pulmonary TB (Dartois, 2014). In addition, these compounds should be screened in combination with other anti-TB and -HIV drugs to better assess efficacy and side effects. Overall, their encouraging *in vitro* and *in vivo* properties uncovered here coupled with their inexpensive production costs (<10 $/kg) make these compounds potentially viable agents for anti-TB therapy (Beteck et al., 2016).

## Author Contributions

LT, RH, and LW were responsible for the conceptualization and design of the study. RH developed and supplied the compounds for testing. LT performed the experiments, analyzed and interpreted the data, drafted the manuscript, and developed the figures and tables. All authors were involved in revising and approved the final version of the manuscript.

## Conflict of Interest Statement

The authors declare that the research was conducted in the absence of any commercial or financial relationships that could be construed as a potential conflict of interest.

## Footnotes

1 Haynes, R. K. South African Medical Research Council Flagship Project North-West University “Development to the Clinical Phase of Oxidant and Redox Drug Combinations for Treatment of Malaria, TB, and Related Diseases”; Available at: http://www.mrc.ac.za/content/strategic-research-initiatives (accessed October 18, 2018).
