# Supplementary material for: An in vitro ADME and in vivo Pharmacokinetic Study of Novel TB-Active Decoquinate Derivatives
Source: Front Pharmacol. 2019 Feb 18;10:120. doi: 10.3389/fphar.2019.00120 (PMC6387968; doi:10.3389/fphar.2019.00120)
Supplement: Supplementary file 1 [file Data_Sheet_1.docx]

An *in vitro* ADME and *in vivo* pharmacokinetic study of novel TB-active decoquinate derivatives

Running title: DMPK study of decoquinate derivatives

**Lloyd Tanner^1^, Richard K. Haynes^2^, Lubbe Wiesner ^*1^**

1. Division of Clinical Pharmacology, Department of Medicine, University of Cape Town, Observatory, 7925, South Africa.
2. Centre of Excellence for Pharmaceutical Sciences, Faculty of Health Sciences, North-West University, Potchefstroom, 2520, South Africa.

**Correspondence:**

*Corresponding author: Associate Professor Lubbe Wiesner

Email: lubbe.wiesner@uct.ac.za

**1. Supplementary material**

**1.1 LC-MS/MS method validation**

Partial method validation, comprising several tests to determine whether the compounds were stable in each possible experimental situation involved with sample collection, storage, and processing, was performed. These tests included: (i) compound stability in mouse whole-blood (on ice and at -80°C), (ii) compound stability in the autosampler, (iii) compound stability in oral and intravenous formulations, (iv) matrix effects (ME), and (v) compound recovery. In this study, stability is reported as a percentage, reflecting the stability of the compound in relation to a fresh sample prepared just before analysis.

The compound concentrations measured in these samples were within 20% range of those of freshly prepared samples of the same concentration extracted using ACN containing IS (Table S1).

**Table S1. Stability testing^a^**

| Compound | Murine whole-blood (ice, 2 h) | | Murine whole-blood (-80 °C freeze- thaw) | | Autosampler stability (48 h) | | Oral formulation stability | IV formulation stability |
| --- | --- | --- | --- | --- | --- | --- | --- | --- |
| Concentration | High^b^ | Low^b^ | High | Low | High | Low | High | High |
| RMB 041 | 91.2 | 89.2 | 98.7 | 99.2 | 94.1 | 92.1 | 98.0 | 90.3 |
| RMB 043 | 109.0 | 102.4 | 92.5 | 91.3 | 99.1 | 99.8 | 89.0 | 93.1 |
| RMB 073 | 92.3 | 87.6 | 97.3 | 89.5 | 102.0 | 105.3 | 89.3 | 98.8 |

^a^values presented as percentages compared to that obtained from a freshly prepared sample; ^b^High, 4000 ng/mL; low, 10 ng/mL

ME assessment involved the preparation of a sample that was spiked into a pre-extracted matrix (A). The concentration of this samples was compared to that of a sample spiked directly into injection solvent comprising ACN and water (1:1, v:v) (B). ME were calculated using Equation S1 below. A value < 100% indicates ion suppression while a value > 100% is indicative of ion enhancement. The majority of compounds exhibited ME that deviated by < 15% from the mean peak area of the solvent samples (Table S2), indicating insignificant ME for these compounds.

**Equation S1. ME determination equation**

$\boldsymbol{ME}\boldsymbol{(\%)=}\frac{\boldsymbol{B}}{\boldsymbol{A}}\boldsymbol{X}\mathbf{100}$

Where A is the pre-extracted matrix peak area response, B is the peak area response of a sample spiked directly into injection solvent, and ME is expressed as a percentage.

**Table S2. Matrix effect (ME) assessment in murine whole-blood**

| Analyte | Mean ratio of peak areas in murine blood (A) | | Mean ratio of peak areas in solvent (B) | | %ME ^a^ | | Standard deviation of murine blood values | | %CV^b^ | |
| --- | --- | --- | --- | --- | --- | --- | --- | --- | --- | --- |
| Concentration | High^c^ | Low^c^ | High | Low | High | Low | High | Low | High | Low |
| RMB 041 | 5.62 | 0.26 | 5.83 | 0.29 | 96.39 | 89.66 | 0.291 | 0.0045 | 5.18 | 1.73 |
| RMB 043 | 4.21 | 0.17 | 4.32 | 0.19 | 97.45 | 89.47 | 0.402 | 0.0073 | 9.56 | 4.29 |
| RMB 073 | 4.01 | 0.11 | 4.44 | 0.13 | 90.31 | 86.61 | 0.390 | 0.0024 | 9.72 | 2.20 |

^a^Matrix effect%; ^b^Percent coefficient of variation; ^c^high, 4000 ng/mL; low, 10 ng/mL

The percentage recovery from murine whole-blood via precipitation with ACN was calculated according to Equation S2. This assessment involved comparing the concentration of a sample that was spiked into a pre-extracted matrix (A) to that of a sample that was spiked into a specific matrix and subsequently extracted (C). The recovery percentage for the compounds (Table S3) was between 83.33% and 96.75%.

**Equation S2. Recovery percentage**

$$\boldsymbol{Recovery} \%=\frac{\boldsymbol{C}}{\boldsymbol{A}} \boldsymbol{X} \boldsymbol{100}$$

Where A is the pre-extracted matrix peak area response and C is the peak area response of a sample spiked into a matrix sample and then extracted.

**Table S3. Compound recovery from murine whole-blood via precipitation with ACN**

| Analyte | Mean ratio of peak areas in murine blood | | Mean ratio of peak areas in solvent | | Recovery (%) | | Standard deviation of murine blood values | | %CV^a^ | |
| --- | --- | --- | --- | --- | --- | --- | --- | --- | --- | --- |
| Concentrations | High^b^ | Low^b^ | High | Low | High | Low | High | Low | High | Low |
| RMB 041 | 5.42 | 0.27 | 5.83 | 0.29 | 92.97 | 93.10 | 0.112 | 0.006 | 2.07 | 2.22 |
| RMB 043 | 4.18 | 0.18 | 4.32 | 0.19 | 96.75 | 94.74 | 0.148 | 0.008 | 3.54 | 4.44 |
| RMB 073 | 4.29 | 0.10 | 4.44 | 0.12 | 96.62 | 83.33 | 0.326 | 0.003 | 7.59 | 3.00 |

^a^Percentage coefficient of variation; ^b^high, 4000 ng/mL; low, 10 ng/mL
